# A New Augmentation Strategy against Depression Combining SSRIs and the N-terminal Fragment of Galanin (1-15)

**DOI:** 10.2174/1570159X23666241003125019

**Published:** 2024-10-31

**Authors:** Antonio Flores-Burgess, Carmelo Millón, Noelia Cantero-García, Juan Pedro Pineda-Gómez, Marta Flores-Gómez, Zaida Díaz-Cabiale

**Affiliations:** 1 Departamento de Fisiología, Facultad de Medicina, Universidad de Málaga, Campus de Teatinos s/n. 29080 Málaga, Spain

**Keywords:** Depression, augmentation therapy, SSRIs, GAL(1-15), animal models, antidepressants

## Abstract

Depression is one of the most disabling mental disorders, with the second highest social burden; its prevalence has grown by more than 27% in recent years, affecting 246 million in 2021. Despite the wide range of antidepressants available, more than 50% of patients show treatment-resistant depression. In this review, we summarized the progress in developing a new augmentation strategy based on combining the N-terminal fragment of Galanin (1-15) and SSRI-type antidepressants in animal models.

## 1 INTRODUCTION

Depression, or major depressive disorder (MDD), is one of the most disabling mental disorders. According to the last estimates by the World Health Organization [1], in 2020, depression was the second disease with the highest social burden. It accounts for 15% of the disease burden, and in 2030, it will become the leading cause of global incapacity [2]. These estimates have become obsolete after the effects of the pandemic derived from the 2019 coronavirus disease on the general population. Some estimations point out that the global prevalence of MDD has grown by more than 27% in recent years, going from 193 affected to 246 million in 2021 [3]. One of the most consistent and enduring findings in research on MDD is a higher prevalence of MDD in women than in men. This gender difference appears in early adolescence, reaches a rate of approximately 2:1 by mid-adolescence, and persists at least through the end of midlife [4, 5]. These differences even tripled the instances with regard to severe depression. Gender differences in rates of MDD have been found cross-culturally and cannot be accounted for by differences in treatment-seeking [6].

The onset of depression is usually gradual for most patients, and the course of the illness is episodic with a duration and pattern variable throughout the lifetime. The diagnosis is determined based on the two central diagnostic classification systems, the Statistical Manual of Mental Disorders (DSM-V) [7] and the International Classification of Diseases (ICD) [8]. This complex emotional disorder is characterised by at least one episode of at least two weeks duration where five or more of the following symptoms are identified: hopelessness and anhedonia as fundamental symptoms, considerable loss or gain of weight and appetite, insomnia or hypersomnia, fatigue or loss of energy and locomotor activity, recurring feelings of worthlessness and guilt, inability to think and indecision, recurring thoughts about death and suicidal ideation with or without an action plan. These episodes are classified based on the quantity and intensity of the symptoms as mild, moderate or severe [7, 8]. In addition to these symptoms, a series of specifiers are defined, especially when MDD is accompanied by anxious distress, the risk of suicide in patients increases, a longer duration of the illness and a greater probability of lack of response to treatments [7]. The MDD also presents a high comorbidity with other types of mental disorders such as panic disorder, obsessive-compulsive disorder, and anorexia nervosa [7] and also with the abuse of substances such as alcohol [9]. Authors have hypothesised that MDD aetiology results from complex interactions of social, psychological, and biological factors during neurodevelopment and beyond [10].

Accompanying this complexity of symptoms and aetiology are numerous pathophysiological alterations related to MDD. Extensive research with animal models and clinical studies has attempted to clarify the neurobiology of depression. Over the years, several hypotheses have been proposed about the cellular and molecular mechanisms underlying MDD and the brain circuits involved. Nevertheless, despite tremendous progress in neuroscience research over the past few decades, the pathophysiology of MDD has yet to be fully elucidated [11, 12].

Between these alterations, the related to monoaminergic systems have received particular attention as a mechanism underlying the symptoms of MDD [13, 14], being that the monoaminergic hypothesis of depression is the oldest to explain the pathophysiology of MDD. Besides, several brain regions and circuits regulate emotion, reward and executive function, and dysfunctional changes within these highly interconnected ‘limbic’ regions are implicated in depression and antidepressant mechanisms [13, 14]. Monoamine projections from midbrain and brainstem nuclei significantly modulate these forebrain networks. Dopamine from the ventral tegmental area (VTA), serotonin from the dorsal raphe (DR), located in the periaqueductal grey area, and noradrenaline from the *locus coeruleus* (LC) [13, 14]. The monoaminergic hypothesis proposes that the reduced availability of these transmitters results in decreased neurotransmission and impaired cognitive performance, which may lead to depression [13, 14].

Another important MDD hypothesis points to an imbalance in the stress response. Stress activates the hypothalamic-pituitary-adrenal axis (HPA) to restore homeostasis, allowing the coordination of brain and body functions that are geared towards coping with stress. Recovery and adaptation to traumatic experiences in early life or acute and chronic stressors challenge the capacity of an individual to cope. If coping fails, various events result in a stable state of distress, reflected in aberrant HPA axis activity and altered limbic function [15-17].

Research has implicated several mechanisms, in addition to the HPA axis and monoamine abnormalities, related to MDD. For example, decreased neuroplasticity and neurogenesis [11, 18], increased inflammatory factors [19], and alterations in the glutamatergic and GABAergic systems [20] and neuropeptides [21, 22]. For more information on the different hypotheses of the pathophysiology of MDD, consult the available literature.

The variety of symptoms and physiological alterations related to MDD allows us to suggest that no single hypothesis explains all aspects of the signs and symptoms of depression. Most probably, depression involves multiple interlinked disease mechanisms that manifest as the constellation of signs and symptoms that depict depression [13]. All this complexity associated with MDD explains the various treatments and pharmacological strategies that have recently emerged and that act on different biological targets. Moreover, the study of the mechanisms of action of antidepressants has revealed new alterations and molecular mechanisms related to the pathology of depression.

## 2 PHARMACOLOGICAL TREATMENTS OF MDD

The first drugs used to treat MDD symptoms, discovered in the 1950s and 60s, were tricyclic antidepressants (TCAs), such as chlorpromazine, and monoamine oxidase inhibitors (MAOIs), such as iproniazide. These drugs are known as first-generation antidepressants and act primarily by raising the monoamine levels in the synapse’s cleft. The main problem with these antidepressants was the elevated number of unwanted side effects, such as cardiovascular effects and relatively high suicide attempts [23, 24].

In the 1980s, the second generation of antidepressants emerged. The first of this second generation was the selective serotonin reuptake inhibitors (SSRIs). These drugs were much more specific to monoaminergic reuptake than TCAs, so they significantly reduced the most harmful side effects [23, 24]. Subsequently, different families of antidepressants have emerged, such as serotonin and norepinephrine reuptake inhibitors (SNRIs), noradrenergic and selective serotonergic antidepressants (NaSSA); serotonin, norepinephrine, and dopamine reuptake inhibitors (SNDRI) or triple reuptake inhibitors (TRI) among others whose pharmacological targets were mainly focused on elements of the monoaminergic systems [24]. It is interesting to note that, despite the large number of antidepressants produced whose molecular target is included within the monoaminergic systems, some of the SSRIs, such as Fluoxetine (FLX), continue to be prescribed today due to their high adherence and safety rates [25].

Nevertheless, one of the main well-known limitations of these drugs is the delay in the onset of therapeutic effects [26]. Different investigations have indicated that the effects of these antidepressants require long-term adaptive changes, in addition to the elevation of monoamine levels in synapses, both at the level of receptors and in signalling cascades.

In the case of SSRIs, the importance of 5-HT1A serotonin receptors (5-HT1AR) has been well characterised both at the autoreceptor level [27, 28] and in the regions of projection [29]. In such a way, the antidepressant effects of these drugs would pass through the autoreceptors' desensitisation at a raphe level [26]. Other modifications that have been described related to the mechanisms of action of the SSRIs and the pathophysiology of depression involve elements of the signalling cascade such as the cAMP-response element binding protein (CREB) [30], regulators of gene expression such as the brain-derived neurotrophic factor (BDNF) [31] and elements that modify the strength and efficiency of synapses in regions such as the dentate gyrus of the hippocampus [32].

More recently, in the 2000s, a new type of antidepressant known as a Rapid-acting antidepressant emerged, which mainly acts by modulating the glutamatergic synapse as NMDA antagonists or by enhancing the function of AMPA receptors [25, 33]. Among these new antidepressants, ketamine is well known, and more recently, psychedelic drugs such as psilocybin, lysergic acid diethylamide, and ayahuasca. However, the mechanisms of action of rapid-acting antidepressants are not fully understood, and the primary impediment to generating a medicine of this type for depressed patients is the potential for side effects [33].

Despite the wide range of antidepressants available on the market or under development, one of the main problems with pharmacological treatments for depression consists of their low efficacy. More than 50% of affected patients do not show improvement after being treated with two or more drugs or with psychotherapeutic interventions, which is called treatment-resistant depression (TRD) [34, 35].

Pharmacological approaches to try to solve TRD involve several strategies: optimization, substitution, and combination or augmentation strategies [36-38]. Substitution strategies, as their name suggests, involve changing the first antidepressant for another, if possible, within the same class, with SSRI-type antidepressants such as FLX being the first to be recommended for treating MDD. This approach has the advantage of avoiding polypharmacy and the appearance of more side effects. However, this strategy also has limited effectiveness [36, 38]. Combination therapies, for their part, have had better results and consist of the co-administration of two antidepressants of the same or different class [36, 38].

Finally, there are augmentation therapies in which an antidepressant is co-administered with a potentiating agent that is not an antidepressant. Included in this group are, for example, potentiating agents such as lithium or thyroid hormones. They have also been tested with beta-blocking SSRIs such as Pindolol, a partial agonist of the serotonin 5-HT1AR [39]. Olanzapine, an antipsychotic with an affinity for the serotonergic 5HT2 and dopaminergic D2 receptors, has also been tested with FLX, obtaining good results in patients with TRD [36, 37]. Other SSRIs, such as Escitalopram (ESC), have been used in augmentation therapies with aripiprazole [40, 41] or buspirone [42].

In this context, studies suggest the implication of several neuropeptides in the pathophysiology of depression, like the urocortin or opioid peptide families, neuropeptide Y, neuropeptide S, Oxytocin, and Vasopressin between others [21, 22]; and point out the possibility of using them as a combined treatment with antidepressant drugs to improve their effectiveness. The neuropeptide Galanin (GAL) is of particular interest in this scenario since GAL is coexpressed in serotonergic neurons of the DR [43, 44], and its receptors are located in key regions in the pathology of depression, such as the limbic system and the prefrontal cortex, interacting and modulating serotonergic receptors.

Besides, and based on Professor Fuxe’s theories about the interactions in G protein-coupled receptor (GPCR) [45, 46], over the last decades, serotonin hetero and isoreceptor complexes in the brain have given new targets for drug development in depression. Specifically, the 5HT1A protomer remains a receptor-enhancing antidepressant action by participating in receptor complexes with different neuropeptide receptors [46], as the heteroreceptor complex from between the fibroblast growth factor receptor and 5HT1A or isoreceptor complexes such as 5HT1A-5HT7 and 5HT1A-5HT2A.

Therefore, the interactions of GAL and GAL(1-15) with the serotonergic system open the possibility of designing new antidepressant drugs by combining endogenous substances with SSRI-type antidepressants where molecular mechanisms complementary to those induced by them are activated. In the following section, we will develop the role of GAL and its N-terminal fragment GAL(1-15) on mood regulation in rodent studies and the interaction between the serotonin and galaninergic systems.

## 3 GALANIN AND IT´S N-TERMINAL FRAGMENT GAL(1-15)

GAL is a 29 amino acid peptide with a highly conserved sequence between species at the N-terminus [47, 48]. It is widely distributed in the central nervous system (CNS), located among other regions in the cerebral cortex, the hippocampus, and the brainstem, where it is synthesized in the noradrenergic neurons of the LC and the serotonergic neurons of the DR [43, 44, 49].

GAL is associated with several physiological functions, such as the central control of food intake, alcohol intake, pain threshold control, cognitive performance, cardiovascular functions, energy homeostasis, neuroendocrine functions, regulation of inflammatory processes, and mood regulation [50-53]. Three types of G protein-coupled receptors have been described whereby GAL mediates its biological activity (GALR1-3 receptors) [54]. GALR1 and GALR3 mainly activate inhibitory G proteins G_i/0,_ while GALR2 is primarily coupled to G_q/11,_ promoting excitatory signals [53]. These receptors are also widely distributed in different brain regions, often overlapping [43].

The role of GAL in depression has been studied using animal models and behavioural tests such as the forced swimming test (FST), a widely validated test for the study of the effects of antidepressants [55, 56], and in animal genetic models [57]. GAL induces pro-depressive behaviour in animal models in the FST through GALR1 or GALR3 receptors [58-60], while activation of GALR2 by specific agonists induces an antidepressant effect [59]. GAL also interacts with the serotonin system at several levels, administered into the lateral ventricle (i.c.v.), can reduce the serotonin (5-HT) metabolism in ventral limbic cortex, hippocampal formation, and fronto-parietal cortex probably *via* direct inhibitory actions on dorsal raphe (DR) 5-HT nerve cells, thru the opening of the GIRK channel [61], reducing their firing rates [62]. At the receptor level, the interaction between GAL and serotonin systems is mediated by the GALR1 and 5HT1AR heteroreceptor complex in discrete brain regions [63], modulating the density or the affinity of the 5HT1AR [61, 64-66].

GAL's effects on anxiety are complex. Studies in rodents indicate that it can have anxiogenic or anxiolytic effects depending on the brain nucleus where it is administered and the behavioural test used [67]. For example, the effects of GAL administered on the caudal raphe nuclei induce a GALR1-mediated anxiogenic effect [68, 69]. At the same time, the administration of the peptide in the dorsal hippocampus produces an anxiogenic effect mediated by GALR2 [70]. GAL interacts with norepinephrine to inhibit LC neurons [71, 72], possibly by interacting at the receptor level with the autoreceptors α2 [72] and is also expressed in hypothalamic neurons that secrete CRF and vasopressin, peptides involved in the regulation of the hypothalamic-pituitary-adrenal (HPA) axis [73]. When GAL is administered directly to the hypothalamus, a decrease in activation of the HPA axis is produced in response to stress [74].

### 3.1 The N-terminal Fragment GAL(1-15)

In addition to GAL, fragments derived from the N-terminus of the peptide, such as the GAL(1-15) fragment, have biological activity and mechanisms of action different from GAL [51, 75]. Although the GAL(1-15) has less affinity for the three GAL receptors than the whole peptide, experiments carried out with the ^125^I-labeled fragment have revealed the presence of binding sites in the CNS with higher affinity for the fragment, as the neocortex, dorsal hippocampus, neostriatum, hypothalamus, and brainstem regions such as the dorsal part of the nucleus tractus solitarius [76]. Many of these regions belong to circuits that regulate emotions, and they are related to depression and antidepressant action. Also, GAL(1-15) has been shown to have biological activity in the regulation of cardiovascular control [51, 77], mood regulation [78, 79], and alcohol intake [80-82].

Concerning mood regulation, studies were carried out to analyse the effects of GAL(1-15) in different behavioural tests in rats validated to analyse the so-called endophenotypes associated with anxiety and depression-like behaviours [83], focusing on anhedonia and despair since they are considered to be a core feature of MDD [7]. In the FST, when an increase in immobility time is associated with the learned despair behaviour in rats, GAL(1-15) induced a more robust depression-like behaviour than GAL. These effects were also observed in the tail suspension test [78]. In the same study, the anxiogenic profile of the fragment was analysed using the open field test (OF) and light-dark box. In both tests, GAL(1-15) modified the anxiety parameters, while GAL did not [78] (Table **1**).

The role of galaninergic receptors in mediating GAL(1-15) behavioural effects was analysed since previous hypotheses have suggested that preferring sites for GAL(1-15) may be formed through the formation of GALR1/GALR2 complex, leading to conformational changes in their recognition sites increasing the affinity for fragment instead GAL [84, 85]. This hypothesis has recently been supported by works using co-transfected HEK cells and Bioluminescence Resonance Energy Transfer (BRET) techniques, giving evidence of the formation of these heteroreceptor complexes [86]. In the same work, differences in signalling cascades between GAL(1-15) and GAL were observed in co-expressing GALR1 and GALR2 HEK cells [86].

**Fig. (1):**
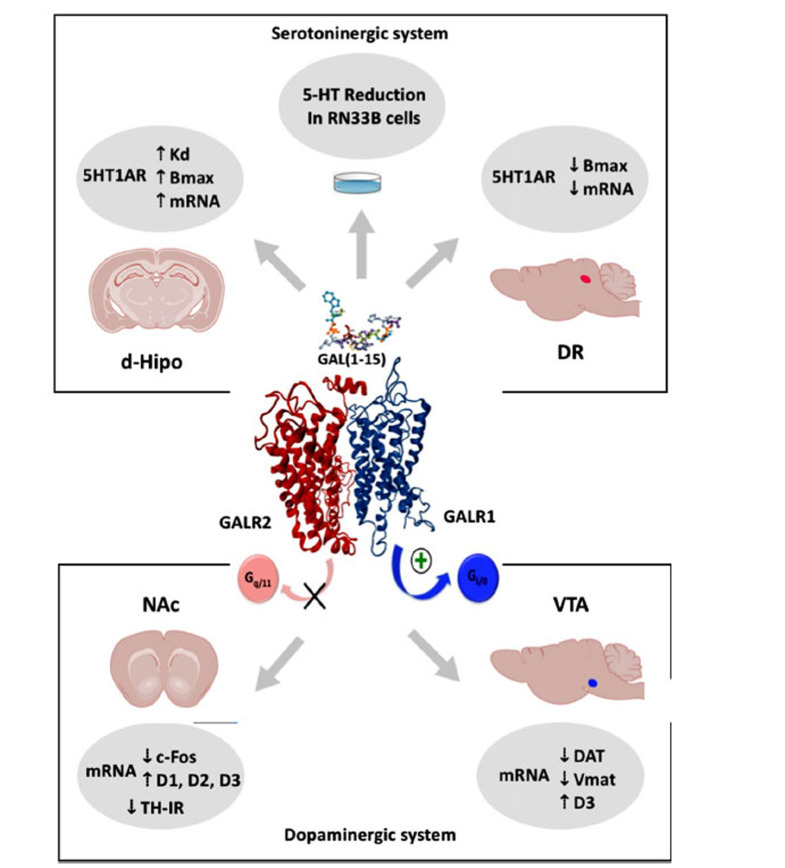
Schematic representation of GAL(1-15) neurochemical effects on serotoninergic (up) and dopaminergic (down) systems mediated by the proposed GALR1-GALR2 heterodimer. GAL(1-15) induces a decrease in the serotonin (5-HT) liberation in RN33B cells and modifies the binding characteristics of 5HT1AR in DR and the dorsal hippocampus (d-Hipo). The fragment modifies the mRNA expression of different dopaminergic system elements in the Ventral tegmental area (VTA) and Nucleus accumbens (NAc). GAL(1-15) also decreases the tyrosine hydroxylase immunoreactivity (TH-IR) in the striatum.

The GALR2 antagonist M871 and knockdown rats for GALR1 (siGALR1) and GALR2 (siGALR2) were used [87]. These knockdown rats were induced by gene silencing by RNA interference specifically designed to reduce the levels of mRNA of both receptors and, therefore, their protein levels [87, 88]. In FST, OF, and SSAT, we observe that GAL(1-15) effects were blocked when coadministered with M871 or in siGALR2 animals [78, 79] (Table **1**). Moreover, GAL(1-15) effects were also blocked in the FST and OF in siGALR1 animals [78]. These results show the critical role of both receptors GALR1 and GALR2 in the behavioural effects of GAL(1-15). On the other hand, in Proximity Ligation *in situ* assays (PLA) performed in raphe-derived RN33B cells and hippocampus and DR tissue sections rats, we observed that GALR1 and GALR2 are close in a range compatible with the formation of GALR1/GALR2 complex [78]. PLA-positive clusters were reduced in the hippocampus and DR of siGALR1 and siGALR2 rats [78], thus relating the decrease of both receptors independently with the formation of heterodimers and the mediation of the behavioural effects of GAL(1-15). In addition to proximity studies of the receptors, in RN33B cells, the administration of GAL(1-15) induced a more potent reduction of the serotonin release than GAL [78] (Fig. **1**), indicating a possible mechanism contributing to the depression-like actions of GAL(1-15). Although previously described as GALR2 may induce antidepressant effects *via* G_q/11_ receptor signalling, it seems possible that in GALR1/ GALR2 heteroreceptor complex GALR1 promoter activate a different signals pathway, or GALR1 promoter could inhibit G_q/11_ mediated-signalling by allosteric receptor-receptor interactions [78, 86] explaining how GAL(1-15) induce a more robust action than GAL.

All these data confirm that GAL(1-15) exerts its actions *via* the formation of the GALR1/GALR2 heterodimer in regions related to depression and shows the differential role of the fragment GAL(1-15) concerning the whole peptide GAL in depression.

Based on this, it was decided to study the possible interaction of GAL(1-15) with the serotoninergic system, specifically with the 5-HT1AR, one of the most studied serotonergic receptors concerning MDD [89]. For this, it was used the 5-HT1AR agonist 8-OH-DPAT [90], which has been shown to have antidepressant effects on FST in rodents [91]. Surprisingly, despite the pro-depressive GAL(1-15) effects described above, the coadministration of GAL(1-15) thresholds dose with 8-OH-DPAT results in an increased antidepressant effect in the FST in rats, with a reduction of 50% in the immobility time and an increase of swimming time of the 80% compared with the groups of animals injected alone with 8-OH-DPAT or GAL(1-15) [92] (Table **1**). This interaction was blocked by the GALR2 antagonist M871, showing the critical role of these receptors in it. Compared to the whole peptide, although GAL also induced a similar effect when co-administered with OH-DPAT, the effects of GAL(1-15) were significantly more robust [92].

In this work, the mechanism of action involved in these behavioural effects was also studied by performing an autoradiography experiment with 5-HT1AR agonist, [H^3^]-8-OH-DPAT, and an *in situ* hybridization to analyse the effects of an effective dose of GAL(1-15) in the binding characteristics and mRNA expression levels of 5-HT1AR in the DR and dorsal hippocampus [92]. Interestingly, GAL(1-15) produced a time-dependent modulation of 5-HT1AR postsynaptically and at the autoreceptor level in the DR. Thus, K_d_ and B_max_ increased from 10 minutes to at least two hours after GAL(1-15) administration in the CA1 and dentate gyrus (DG) areas of the dorsal hippocampus join with an increase of mRNA levels of 5-HT1AR two hours after the administration [92] (Fig. **1**). In the DR, we observed a reduction of B_max_ and mRNA levels but only at two hours after the administration of GAL(1-15) [92] (Fig. **1**). These effects are also different from those that the complete peptide induces on 5-HT1AR, as we saw in previous studies, where GAL lacks effects on the [H^3^]-8-OH-DPAT binding characteristics in the hippocampus [64, 93] and on the contrary, in the DR, a time-dependent reduction in affinity and an increase in the 5-HT1A autoreceptor density was observed after GAL treatment [64]. The GAL-mediated effect in the limbic forebrain and also in the DR was explained due to the existence of GALR1/5-HT1AR heteroreceptor complexes demonstrated in cellular models [63], within them antagonistic allosteric receptor-receptor interactions which counteract the 5-HT1AR induced activation of G_i/o_-mediated signalling at least in specific 5-HT1AR signalling cascades [63, 92].

Together with the ability of GAL(1-15) to modify the binding characteristics and mRNA expression levels of 5-HT1AR, and using PLA techniques, it was seen that is not only GALR1 near 5-HT1AR but also GALR2 in a range compatible with the formation of GALR2/5-HT1AR complex in both DR and dorsal hippocampus [92]. Since in previous work, it was demonstrated that the heteroreceptor complex GALR1/GALR2 is the biological target of GAL(1-15) [78], these PLA results support the possible existence of GALR1/GALR2/5-HT1AR complexes in DR and dorsal hippocampus [92]. These heteroreceptor complexes could work as integrative nodes in serotoninergic neurotransmission and as significant targets for the antidepressant effects of GAL(1-15) at the autoreceptor and postjunctional level of serotoninergic transmission of the raphe-hippocampal neurons [92], modulating the functionality of 5-HT1AR and the behavioural effects described above.

More recently, the interactions of GAL(1-15) with other monoaminergic systems, such as the dopaminergic system, were also studied. The role of GAL(1-15) was tested in a key endophenotype related to depression as reward-related and anhedonic behaviour using animal models in a non-operant test as sucrose preferent test and novelty suppressed feeding and female urine sniffing test and in an operant test as saccharine self-administration test (SSAT) [79] (Table **1**). In all these tests, it was seen that the fragment induced a strong anhedonia-like phenotype compared with GAL [79]. Using PCR and immunohistochemistry techniques, it was seen that GAL(1-15) induced a decrease in the mRNA expression of the dopamine transporters Dat and Vmat in the ventral tegmental area (VTA) and c-fos mRNA expression levels in the nucleus accumbens (NAc) join together with an increase of the mRNA expression levels of the D1, D2 and D3 dopamine receptors in NAc and D3 in VTA [79] (Fig. **1**). GAL(1-15) also induce a decrease in the immunoreactivity of TH in the striatum (CPu) and NAc regions, being more intense in the core of the NAc [79]. VTA and NAc are the key nuclei of the dopaminergic mesolimbic circuit. All these data point out that the role of GAL(1-15) is critical for the neurobiological bases of anhedonia, a core feature of MDD.

In addition to the effects of GAL(1-15) described in the different brain nuclei, PET imaging was carried out using ^18^F-fluorodeoxyglucose (^18^F-FDG) to analyse the ^18^F-FDG uptake between GAL(1-15)-injected animals and controls *in vivo*. This experiment reveals that GAL(1-15) decreases the ^18^F-FDG uptake in the hippocampus, thalamus, and striatum. Moreover, it also increases ^18^F-FDG uptake in the prefrontal and piriform cortex [79]. The effects of GAL(1-15) in the ^18^F-FDG uptake are in agreement with previous works in which the hippocampus or the striatum were targeted for GAL(1-15) [78-80, 87], which are areas rich in GAL-fragment binding sites [76].

## 4 AUGMENTATION THERAPY WITH GAL(1-15) AND SSRIS

As described in the previous section, the GAL(1-15) interacts with serotoninergic systems [79, 92] at behavioural and receptor levels, proposing the existence of GALR1/ GALR2/5-HT1AR complexes as integrative nodes in serotoninergic neurotransmission in the DR and dorsal hippocampus regions [92].

These brain regions have been previously studied for the antidepressant effects of SSRIs, describing essential changes related to their chronic administration [26, 28, 29]. Therefore, we decided to explore the interaction between GAL(1-15) and SSRI-type antidepressants since numerous studies highlight the crucial role of the 5-HT1AR and their modulation in the mediation of the SSRI’s antidepressant effects [26-29].

### 4.1 Interactions of GAL(1-15)-SSRIs in Rats

Between all the different SSRI-type antidepressants, first it was tested the potential interaction of GAL(1-15) with FLX for several reasons. Although it was one of the first SSRIs to be developed, today, it continues to be prescribed due to their high adherence and safety rates [25], being one of the most used antidepressants in augmentation therapies and even recommended with cognitive-behavioural therapy, as it offered the most favourable tradeoff between benefit and risk for adolescents with MDD [94]. In addition, it is one of the best-studied and characterized antidepressants in several animal models, including the role of 5-HT1AR in mediating its effects.

The coadministration of GAL(1-15) with FLX at threshold doses in rodents resulted in an antidepressant-like effect in the FST with a decrease in immobility time and an increase of the swimming time, indicating a pharmacological interaction between the two compounds that enhances the effects of FLX as we expected [95]. This interaction was confirmed with an effective dose of FLX where GAL(1-15) enhanced the antidepressant effects of FLX by 50% in the FST [95]. These effects were not obtained when GAL was used in combination with FLX, showing the differential behaviour effects of GAL(1-15) compared to GAL again (Table **1**).

The role of GALR1 and GLAR2 was critical in the enhancing effect of GAL(1-15) on FLX since using knockdown rats for both receptors was able to block the decrease of immobility time and the increase of swimming time induced by the coadministration of GAL(1-15)+FLX in the FST [95] (Table **1**), reinforcing the previous hypothesis that the heterodimer GALR1/GALR2 is the molecular target for GAL(1-15) were allosteric interactions inhibit the Gq/G11-mediated signalling of the GALR2 protomer and switches the isoreceptor complex towards Gi/o-mediated signalling [78, 86].

The role of 5-HT1AR in the interaction was also analysed in a behavioural test in this study, using the antagonist Way100635 in a pattern of administration that not interferes with FLX effects [96, 97]. The antagonist blocked the enhancing effect of GAL(1-15) on FLX without affecting the effects of FLX [95] (Table **1**), indicating the key role of 5-HT1AR in the interaction. This result is highly interesting since it supports the notion that GAL(1-15) through agonist activation of allosteric GALR1/GALR2/5-HT1AR receptor-receptor interactions in putative trimeric heteroreceptor complexes may include effects on the recognition, pharmacology and signalling of the participating 5-HT1AR protomers. In this way, when FLX induces an increase in serotonin's extracellular levels, it may also favour the formation of this putative higher-order GALR1/GALR2/5-HT1AR heteroreceptor complexes. In such a complex, altered allosteric receptor-receptor interactions can develop with an ability of the GALR1/GALR2 component to enhance the 5-HT1AR protomer signalling.

**Fig. (2):**
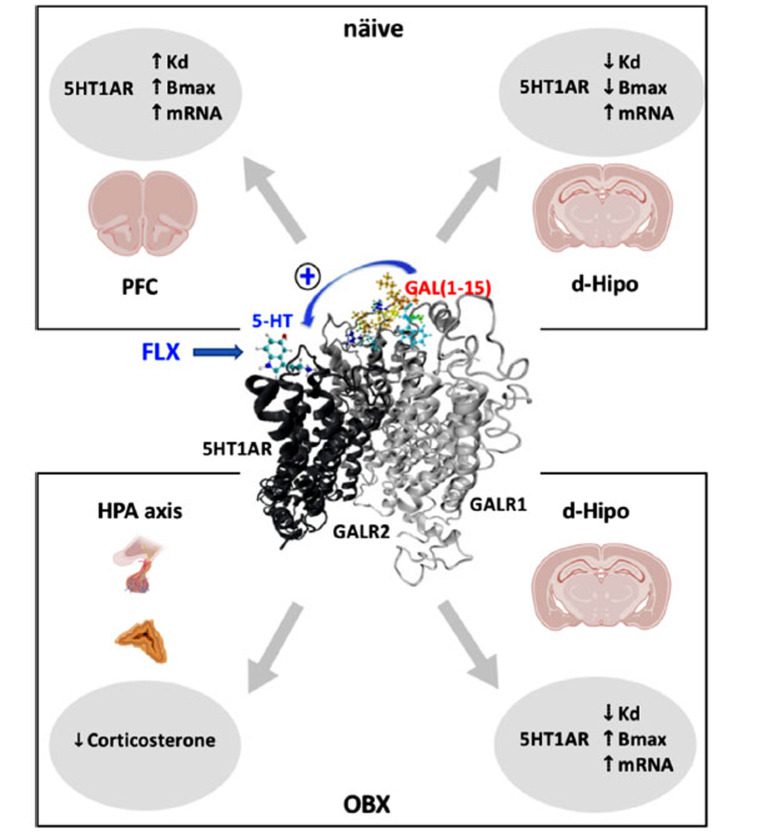
Schematic representation of the molecular mechanics underlying the GAL(1-15)+FLX effects in näive (up) and bulbectomized animals (OBX) (down). The receptor complex GALR1/GALR2/5HT1AR is proposed as the molecular complex where 5HT1AR binding properties are modified differently in the Prefrontal cortex (CPF) and dorsal hippocampus (d-Hipo). The effects in the HPA axis is also described.

The mechanism of action involved in the pharmacological combination of GAL(1-15) and FLX was studied by performing an autoradiography experiment with the 5-HT1AR agonist, [H^3^]-8-OH-DPAT, and an *in situ* hybridisation to analyse the effects of the combination of GAL(1-15)+FLX in the 5-HT1AR binding characteristics and mRNA expression levels in several brain nuclei [95]. In these experiments, a reduction in K_d_ and Bmax and increased mRNA levels of postjunctional 5-HT1AR in the dorsal hippocampus, specifically in the DG, were observed. No effects were observed in the DR on the binding characteristics or expression of soma-dendritic 5-HT1AR autoreceptors [95] (Fig. **2**). This effect points out again to the existence of GALR1/GALR2/5-HT1AR heteroreceptor complexes where allosteric interactions modify the binding characteristics of 5HT1AR protomer in the DG and shows the essential role of 5-HT1AR in the GAL(1-15)-FLX interaction. Nevertheless, it is not possible to exclude that a potential enhancement in the firing rate of the ascending serotoninergic DR neurons was induced by GAL(1-15) by counteracting the serotonin-induced autoreceptor signalling despite the lack of effects on 5-HT1AR autoreceptor recognition since we only analyse the binding characteristics of mRNA in one temporal point.

The behavioural interaction of GAL(1-15) with FLX in behavioural learning paradigms was also tested since 5-HT1AR has been related to one of the serotoninergic receptors involved in the neurobiological control of learning and memory [98]. It is also interesting to note that, in addition to the emotional symptoms related to depression, numerous studies indicate that there are cognitive deficits in depressed patients and even that these deficits can be a predictor of patients who do not respond to treatment with SSRIs such as FLX [99, 100].

The Object Location Memory (OLM) and the Novel Object Recognition (NOR) tests were used since the hippocampus is a crucial region in spatial memory tasks such as the OLM [101]. At the same time, the NOR is an associative learning task based on natural exploration where the medial prefrontal cortex (mPFC) has a crucial role [98, 102]. In both tasks, FLX treatment did not affect learning or short-term memory, but specifically impaired long-term memories since animals did not explore the new objects in the test session performed 24h post-training either in the OLM or in the NOR task [103, 104].

In these experiments, the combination of GAL(1-15)+FLX in rats did not change the parameters analysed in the OLM compared to FLX, while in the NOR, GAL(1-15) reversed the effects of memory impairment induced by FLX. This effect was blocked by the GALR2 antagonist M871 [105] (Table **1**), revealing the key role of this receptor in this effect. The results in the NOR cannot be explained by the modulation of other memory phases, such as the acquisition or the retention due to the pattern of administration of substances, suggesting that GAL(1-15) is acting through the mPFC in order to modulate memory retrieval [106, 107].

To delve into these behavioural results, it was analysed the effects of the GAL(1-15)+FLX in the functional characteristics of the 5-HT1AR in the medial prefrontal cortex (mPFC), a core region for the interaction between emotional processing and cognition [108]. Contrary to what we observed in the dorsal hippocampus, the GAL(1-15) + FLX combination led to an increase in both Kd and Bmax. In contrast, again, an increase in 5-HT1AR mRNA expression was also observed in this region [105] (Fig. **2**). These results could indicate that the molecular complexes GALR1/ GALR2/5-HT1AR are likely highly dynamic and vary from one brain region to another as a consequence of differences in the composition of the 5-HT1AR heteroreceptor complexes involved in the two regions as the adapter proteins or differences in the coupling of 5-HT1AR to G protein since 5-HT1AR interact equally with G_α0_ and G_αi3_ in the cerebral cortex but mainly with G_αo_ and weakly with G_αi3_ in the hippocampus [109].

The results in the NOR and OLM tasks could be associated with the proposed trimeric GALR1/GALR2/5-HT1AR heteroreceptor complex in both areas of the hippocampus and mPFC. In the OLM, results agree with previous data from our group demonstrating that the GALR1/GALR2/5-HT1AR heteroreceptor in the hippocampus enhances 5-HT1AR signalling in the complex by, inter alia, reducing 5-HT1AR internalization from the plasma membrane. In the (NOR) test, GAL(1-15) may impair 5-HT1A receptor signaling through the GALR1/GALR2 heteroreceptor complex within the trimer, which explains why coadministration of GAL(1-15) and FLX reversed the effects mediated by FLX alone.

However, to explain these results, we cannot exclude the influence of other serotoninergic receptors or brain areas that regulate emotional and cognitive behaviours. Taking these results together, it can be suggested that the combination of the GAL(1-15) and FLX modulates the serotonergic circuit, or a part of it, in such a way that enhances the antidepressant effects and improves the cognitive impairments induced by FLX.

### 4.2 Interactions GAL(1-15)-ISRSs in Depressive Animal Model

To determine the potential use of GAL(1-15) as an SSRI antidepressant augmentation agent, and once the interaction between GAL(1-15) and FLX was determined, the effects of this pharmacological combination were tested in an animal model of inducible depression, the bulbectomised (OBX) rat. This animal model mimics several symptoms observed in patients with MDD, resulting in an optimal animal model since it is widely described that the bilateral destruction of the olfactory bulbs causes complex alterations in behaviour and both biochemical and cellular mechanisms: anhedonia in sucrose preference, increased hyperactivity in a new environment, reduced sexual activity, and elevated corticosterone levels. This animal model also tested other SSRIs besides FLX in order to analyse if GAL(1-15) could enhance the antidepressant effects of SSRIs. Escitalopram (ESC) is an SSRI more efficacious than FLX in reducing the depressive symptoms for the acute phase treatment of MDD [110] and has been widely studied in augmentation therapy in treatment-resistant depression (TRD) with aripiprazole [41] or buspirone [42].

The behavioural interaction between GAL(1-15) and FLX in the FST was replicated in OBX rats. Again, an antidepressant-enhancing effect was observed mediated by the fragment with increased swimming time in OBX animals administered with the fragment and FLX [111]; the GALR2 antagonist blocked this enhancing effect. It is interesting to note that in this work, it was also determined that the expression levels of the mRNA of the galaninergic receptors involved in the interaction between GAL(1-15)-FLX, GALR1, and GALR2 did not show differences due to bulbectomy [111].

In addition to the FST, the effects of the GAL(1-15)-FLX combination in the Sucrose Preference Test (SPT) were also tested. SPT detects an anhedonia-like condition, which is a core feature of major depression and characteristic of the OBX model [79, 112]. The results in the SPT indicated that GAL(1-15) enhanced FLX-induced antidepressant effects in the FST and restored the anhedonia-like condition observed in the OBX animals that FLX alone could not (Table **1**). All these results confirm a potent effect of the combination GAL(1-15)-FLX in reversed depressive symptoms in depressive animal models at the behavioural level.

The characteristics, functionality, and mRNA expression of the 5-HT1AR after pharmacological treatments were also analysed using autoradiographic techniques with [H^3^]-8-OH-DPAT, *in situ* hybridisation, and specific [^35^S]GTPγS binding in the dorsal hippocampus of OBX animals. The results of these experiments show that bulbectomy does not affect the density or binding characteristics of the 5HT1AR in the hippocampus, as previously described [113, 114]. However, the coadministration of FLX with GAL(1-15) in OBX animals induced an increase in the mRNA levels of 5-HT1AR in the dorsal hippocampus and a remarkable increase in the B_max_ (Fig. **2**), specifically in the DG, suggesting an essential role of hippocampal 5-HT1AR in the GAL(1-15)-FLX interaction. These modifications agree with our previous work in naïve animals [95] and confirm the crucial role of 5-HT1AR in the hippocampus in the GAL(1-15)-FLX interaction reinforcing our previous hypothesis: the existence of a trimeric GALR1/GALR2/5-HT1AR heteroreceptor complex in cortico-limbic areas where altered allosteric receptor-receptor interactions can develop an ability of the GALR1-GALR2 component to enhance the 5-HT1AR protomer signalling [92, 95, 105] and could be the critical point to understand the effects of FLX-GAL(1-15) interaction in the OBX animal depression model.

Besides behavioural and neurochemical effects of the GAL(1-15) and FLX combination in the OBX animals, the corticosterone circulating levels were analysed, and only the coadministration of GAL(1-15)+FLX was able to reduce the OBX-increased corticosterone blood levels by approximately 50% (Fig. **2**). At the same time, FLX treatment did not affect basal corticosterone levels in OBX animals as previously described [111, 115]. This result indicates that the interaction between FLX and GAL(1-15) might involve the regulatory elements of the HPA axis. Preclinical and clinical studies have gathered substantial evidence that hyperactivity of the hypothalamic-pituitary-adrenocortical (HPA) system is one of the significant pathophysiological factors for the development of depression [116]. The long-term administration of antidepressants may normalise this HPA alteration because this effect is associated with antidepressant-induced clinical improvement [111, 117]. However, persistent abnormality of the HPA axis correlates with resistance to treatment or relapse in MDD [118]. Therefore, the fact that the elevated levels of corticosterone, characteristic of OBX animals, are not counteracted with the administration of FLX alone [115] but significantly decrease after the joint administration of GAL(1-15) and FLX indicates that the interaction between both drugs could involve the regulatory elements of the HPA axis and opens the possibility of using this treatment in resistant therapy. Also, the decrease in the corticosterone blood levels observed after the coadministration of GAL(1-15) and FLX could be partly responsible for the increase observed in the mRNA levels and B_max_ of 5-HT1AR in the dorsal hippocampus since one of the most potent regulators of 5-HT1AR expression in the rat brain is corticosterone [111, 117].

Behavioural data obtained in the OBX animal model with the GAL(1-15)+FLX combination was partially reproduced when another SSRI-type antidepressant, ESC, was used. In these experiments, GAL(1-15) enhanced ESC effects in behavioural tests related to despair, the FST, and the tail suspension test (TST) [119]. In FST, the GALR2 antagonist M871 blocked GAL(1-15) mediated actions in the FST, and the downregulation of 5-HT1AR by siRNA was sufficient to block GAL(1-15) enhancement of the antidepressant-like effects mediated by ESC [119] (Table **1**). All these data are in agreement with previous experiments performed with FLX in OBX and naïve animals and confirm a potent effect of the combination of GAL(1-15) with SSRIs in reversed depressive symptoms and open up the possibility to use this combination as augmentation therapy in MDD [95, 105, 111].

**Fig. (3):**
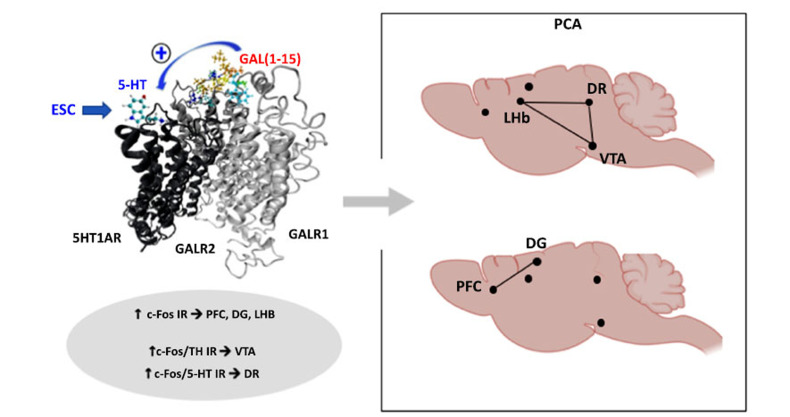
Schematic representation of the proposed neural networks after Principal component analysis (PCA) from the data obtained from the c-Fos immunoreactivity (c-Fos IR) in the Prefrontal cortex (PFC), Dentate gyrus of the dorsal hippocampus (DG) and Lateral habenula (LHb); and double immunoreactivity to c-Fos + Tyrosine hydroxilase (c-Fos/TH IR) in the Ventral tegmental area (VTA) and c-Fos + serotonin (c-Fos/5-HT IR) in the dorsal raphe (DR). The coadministration of GAL(1-15) + Escitalopram (ESC) induces an increase of the c-Fos IR in PFC, DG, and LHb compared to the control group and also an increase of c-Fos/TH IR and c-Fos/5-HT IR in VTA and DR respectively.

In this work, an immunohistochemical study was performed to analyse the activation profile of different brain nuclei using the c-Fos immunoreactivity. The coadministration of GAL(1-15)+ESC in OBX rats produces a significant increase in the number of c-Fos-IR profiles in several nuclei involved in MDD as the dorsal hippocampus, PFC, and Lateral Habenula (LHb) (Fig. **3**). Moreover, an increase in the number of c-Fos-IR serotoninergic cell bodies in the DR and the c-Fos-IR TH cell bodies in the VTA was observed after GAL(1-15)+ESC [119] (Fig. **3**). Posterior principal component analysis (PCA) based on immunohistochemical data indicated two possible functional brain networks opening up the possibility that the participation of different areas is interrelated. One comprised the DG and PFC and the other LHb, DR, and VTA regions.

The involvement of the lateral habenula (LHb) in the functional brain network is of great interest, as the LHb has recently been linked to depressive symptoms such as helplessness and anhedonia [119-121]. In animal models, ketamine, a new rapid antidepressant, acts by suppressing the activity of LHb neurons [119, 122]. Besides, LHb is one of the few brain regions that control both the dopaminergic system and the serotonin system, including the VTA and the DR [120, 121]. In relation to the second network, both DR [78] and VTA [79] have been previously reported to be involved in GAL(1-15)-mediated effect; in fact, we have proposed that the VTA through the VTA-limbic-cortical DA system is responsible for the anhedonia-like behaviour induced by GAL(1-15) [79, 119].

Given the profile of results obtained in the animal models, we consider that an augmentation SSRI treatment with GAL(1-15), by some clinical guidelines, could be appropriate in TRD patients who show anxious distress and cognitive dysfunction among their symptoms and in a first approximation, they have not had good results with the administration of SSRIs alone. Another possible group of patients that could be susceptible to being treated with this argumentation therapy would be patients who present with alcohol use disorder and MDD comorbidity [123].

## CONCLUSION

All these data allow us to suggest that the pharmacological combination of GAL(1-15)+SSRIs could be an effective augmentation therapy that not only enhances the antidepressant effects of SSRIs but also could modulate several systems altered in depressive patients as the HPA axis and several neuronal limbic circuits implicated in depression symptoms as despair and anhedonia, based in the interactions between the galaninergic and serotonergic systems. Furthermore, although it is necessary to expand the studies on the mechanisms of action and the effects in female models of this potential therapy, it could be a promising pharmacological strategy for cases of resistant depression.

## Figures and Tables

**Table 1:** Behavioural effects of the GAL(1-15) administration alone or in combination with the 5HT1AR agonist 8-OH-DPAT or the SSRIs Fluoxetine (FLX) or Escitalopram (ESC) in different behavioural tests.

**Rat Model**	**Treatment**	**Test**	**Effect**	**siGALR1**	**siGALR2**	**M871**	**si5HT1AR**	**Way100635**	**References**
Sprague-Dawley (SD)	GAL(1-15) (1 nmol) subthreshold	FST	0	-	-	-	-	-	[78]
		OF	0	-	-	-	-	-	[78]
		SSAT	0	-	-	-	-	-	[79]
SD	GAL(1-15) (3 nmol) effective	FST	↓	∅	∅	∅	-	-	[78]
		TST	↓	-	-	-	-	-	[78]
		OF	↓	∅	∅	∅	-	-	[78]
		D/L	↓	-	-		-	-	[78]
		SSAT	↓	-	-	∅	-	-	[79]
		SPT	↓	-	-	-	-	-	[79]
		NSF	↓	-	-	-	-	-	[79]
		FUST	↓	-	-	-	-	-	[79]
SD	GAL(1-15) (1 nmol) subthreshold + 8-OH-DPAT (0.125 mg/Kg) subthreshold	FST	↑↑	-	-	∅	-	-	[92]
SD	GAL (1 nmol) subthreshold + 8-OH-DPAT (0.125 mg/Kg) subthreshold	FST	↑	-	-	-	-	-	[92]
SD	GAL(1-15) (1 nmol) subthreshold + FLX (2.5 mg/Kg) subthreshold	FST	↑	-	-	-	-	-	[95]
SD	GAL(1-15) (1 nmol) subthreshold + FLX(10mg/Kg) effective	FST	↑↑	∅	∅	∅	-	∅	[95]
		NOR	↑*	-	-	∅	-	-	[105]
		OLM	↓*	-	-		-	-	[105]
Bulbectomised (OBX)	GAL(1-15) (1 nmol) subthreshold + FLX(10 mg/Kg) effective	FST	↑↑	-	-	∅	-	-	[112]
		SPT	↑	-	-	-	-	-	[112]
OBX	GAL(1-15) (1 nmol) subthreshold + ESC(10 mg/Kg) effective	FST	↑	-	∅	-	∅	-	[120]
		TST	↑↑	-	-	-	-	-	[120]

**Note:** ↓ Depressive or anxiogenic effect; ↑ antidepressant effect; ↑↑ antidepressant enhanced effect; ↓* impaired memory effect; ↑* reversed impaired memory effect; 0 no effect related to the control group. The enhancing effects of GAL(1-15) on antidepressants or alone were blocked (∅) by the administration of antagonists GALR2 (M871) and 5HT1AR (Way100635) or by knock-down rats for GALR1 (siGALR1), GALR2 (siGALR2) and 5HT1AR (si5HT1AR).

## LIST OF ABBREVIATIONS

BDNF: Brain-derived Neurotrophic Factor
CNS: Central Nervous System
DR: Dorsal Raphe
HPA: Hypothalamic-pituitary-adrenal Axis
LC: Locus Coeruleus
MDD: Major Depressive Disorder
OLM: Object Location Memory
SSAT: Saccharine Self-administration Test
SSRIs: Selective Serotonin Reuptake Inhibitors
TRD: Treatment-resistant Depression
TRI: Triple Reuptake Inhibitors
TST: Tail Suspension Test
VTA: Ventral Tegmental Area
